# The Facile Preparation of PBA-GO-CuO-Modified Electrochemical Biosensor Used for the Measurement of α-Amylase Inhibitors’ Activity

**DOI:** 10.3390/molecules27082395

**Published:** 2022-04-07

**Authors:** Min Li, Xiaoying Yin, Hongli Shan, Chenting Meng, Shengxue Chen, Yinan Yan

**Affiliations:** 1 College of Chemistry and Chemical Engineering, Shanghai University of Engineering Science, Shanghai 201620, China; 18516629660@163.com (M.L.); mli19960904@163.com (H.S.); kamiyahc@163.com (C.M.); sxchen1009@163.com (S.C.); 2 School of Chemistry and Chemical Engineering, Institute for Frontier Medical Technology, Shanghai 201620, China; 3 National Engineering Research Center for Nanotechnology, 28 East Jiang Chuan Road, Shanghai 200241, China

**Keywords:** graphene, nonenzymatic glucose sensor, boron-doped, copper oxide

## Abstract

Element doping and nanoparticle decoration of graphene is an effective strategy to fabricate biosensor electrodes for specific biomedical signal detections. In this study, a novel nonenzymatic glucose sensor electrode was developed with copper oxide (CuO) and boron-doped graphene oxide (B-GO), which was firstly used to reveal rhubarb extraction’s inhibitive activity toward α-amylase. The 1-pyreneboronic acid (PBA)-GO-CuO nanocomposite was prepared by a hydrothermal method, and its successful boron doping was confirmed by transmission electron microscopy (TEM) and X-ray photoelectron spectroscopy (XPS), in which the boron doping rate is unprecedentedly up to 9.6%. The CuO load reaches ~12.5 wt.%. Further electrochemical results showed that in the enlarged cyclic voltammograms diagram, the electron-deficient boron doping sites made it easier for the electron transfer in graphene, promoting the valence transition from CuO to the electrode surface. Moreover, the sensor platform was ultrasensitive to glucose with a detection limit of 0.7 μM and high sensitivity of 906 μA mM^−1^ cm^−2^, ensuring the sensitive monitoring of enzyme activity. The inhibition rate of acarbose, a model inhibitor, is proportional to the logarithm of concentration in the range of 10^−9^–10^−3^ M with the correlation coefficient of R^2^ = 0.996, and an ultralow limit of detection of ~1 × 10^−9^ M by the developed method using the PBA-GO-CuO electrode. The inhibiting ability of Rhein-8-b-D-glucopyranoside, which is isolated from natural medicines, was also evaluated. The constructed sensor platform was proven to be sensitive and selective as well as cost-effective, facile, and reliable, making it promising as a candidate for α-amylase inhibitor screening.

## 1. Introduction

The inhibitors of α-amylase can prevent some late complications by suppressing the postprandial rise of blood glucose. The analysis methods applied for α-amylase inhibitor measurement are studied by many researchers [1,2].

Electrochemical sensor strategies are prevailing and deserve more attention due to intrinsic advantages such as high sensitivity and selectivity [3,4,5,6] and direct monitoring of the target enzymes without complicated pretreatment [7,8,9,10]. In the construction of an electrochemical sensor, chemically modified electrodes improve electron transport rate at a low potential, resulting in a decrease in the interference of impurities and an increase in the sensitivity of the current response [11,12]. The performance of the sensors improved due to development of nanotechnology such as the detection limit and a wide range of detection of target molecules. These advantages allow electroanalytical methods to be widely used in the determination of biological and environmental analysis.

Recently, considerable attention has been focused on developing nonenzymatic glucose sensors since it overcomes such drawbacks of traditional enzyme glucose sensors as instability, high cost of enzymes, complicated immobilization procedure, critical operating situations, etc., [13,14,15,16]. This also provided a new idea for identifying and detecting α-amylase inhibitors. Metals alloys, metal nanoparticles, and noble metals have been extended to develop nonenzymatic glucose sensors [17,18,19,20]. However, these electrodes have such disadvantages as high cost, low selectivity, or poisoning of chloride ions, which greatly limit their applications [21,22,23,24,25]. Thus, developing a highly selective, fast, reliable, and cheap nonenzymatic glucose sensor is still imperatively demanded.

Graphene is a well-known conductive material composed of two-dimensional honeycomb lattice-structured carbon atoms connected by an sp² monolayer [26,27,28,29]. Studies have shown that doping heterogeneous elements, such as nitrogen, boron, oxygen, sulfur, and halogens can effectively improve electrochemical performance [30,31,32]. First, boron doping can enhance the contact area between graphene, CuO nanoparticle, and electrolyte [33]. Furthermore, the electron deficiency of the boron element doped on the graphene acts as a superior electron receiver [34]. The materials mentioned above acting as a substrate to support CuO nanoparticles can vastly improve conductivity and sensing ability [35]. On the other hand, as a p-type semiconductor with a narrow bandgap of 1.2 eV, CuO has been widely studied because of its numerous applications in semiconductors, catalysis, biosensors, field transistors, and gas sensors. The CuO nanowires are an important nanoparticle in modifying electrodes with high sensitivity. However, the synthesis of CuO nanowires is tedious and time-consuming [34].

In this study, a novel PBA-GO-CuO nanoparticle was prepared through the hydrothermal method, and the synergistic effect of PBA and GO dramatically improved the electrocatalytic properties of glucose oxidation and detection. The developed detection platform using PBA-GO-CuO nanoparticles provided an acceptable detection limit of 0.95 nM to acarbose at a signal-to-noise ratio of 3, indicating ultra-sensitivity to α-amylase inhibitors. Rhein-8-b-D-glucopyranoside isolated from natural products was screened by the proposed sensing platform, demonstrating the excellent applicability [36]. The correctness was also verified using the iodine assay colorimetric method [37]. The constructed sensor platform was proven to be facile and cost-effective as well as highly sensitive, selective, and reliable, making it promising as a candidate for trace inhibitor screening of natural products.

## 2. Results and Discussion

### 2.1. Characterization of PBA-GO-CuO Nanocomposite

**Figure 1:**
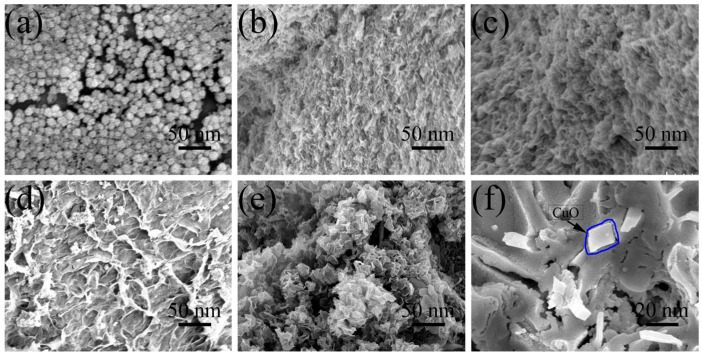
SEM image of (**a**) CuO, (**b**) GO, (**c**) PBA-GO, (**d**) GO-CuO, (**e**) PBA-GO-CuO, (**f**) PBA-GO-CuO.

SEM images of various aggregates are shown in Figure 1, displaying distinct morphologies during the synthesis of PBA-GO-CuO nanomaterials. Figure 1a presents typical ellipsoidal CuO particles with a size of ~23 nm. In Figure 1b, graphene oxide aggregates appeared as ruffled wrinkles abound on surface areas. As shown in Figure 1c, the macroscopic stacked structure becomes predominant after boron doping. Moreover, Figure 1d displays the SEM images of CuO-GO hybrid material. The pronounced aggregates are observed due to the CuO load on graphene oxide [38,39,40]. Figure 1e portrays the PBA-GO-CuO nanomaterials’ rich pore structure and high specific surface area. From the enlarged picture in Figure 1f, we can see with more detailed information that the size of CuO is smaller than that in Figure 1a.

**Figure 2:**
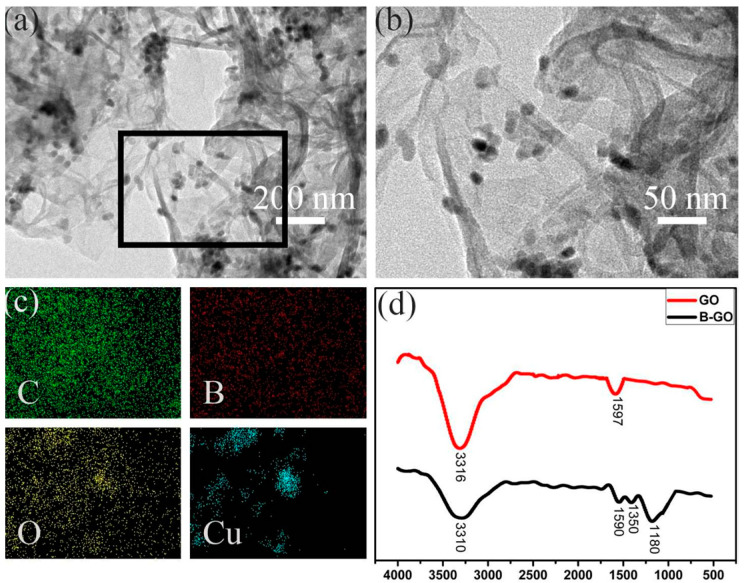
(**a**) TEM image of PBA-GO-CuO, (**b**,**c**) EDS mappings of PBA-GO-CuO for C (green), B (red), O (yellow), and Cu (blue), (**d**) FTIR image.

The TEM and energy dispersive X-ray spectra (EDS) are exhibited in Figure 2. In Figure 2a,b, massive amounts of CuO particles are decorated on the graphene oxide surface, affirming the SEM observation. From the EDS mapping surface scan (Figure 2c), we can see that the elements of carbon (green), boron (red), and oxygen (yellow) are uniformly overlapped, proving once again that CuO particles are homogeneously braced on graphene oxide. The FTIR images in Figure 2d confirm the boron doping with the characteristic absorption bands of B–O at 1350 cm^−1^ and B–C at 1180 cm^−1^ [35].

**Figure 3:**
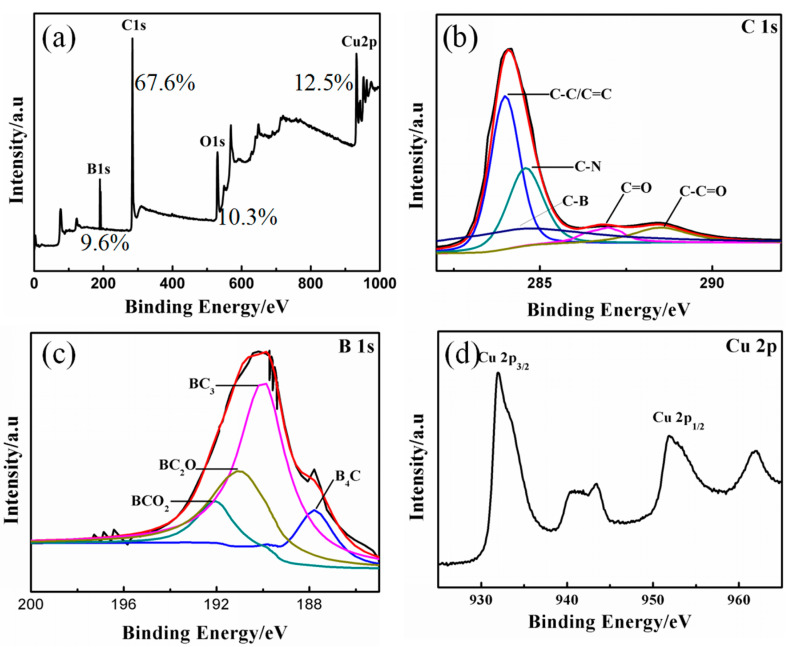
XPS curves: (**a**) whole spectra of PBA-GO-CuO, (**b**) C1s of PBA-GO-CuO, (**c**) B1s of PBA-GO-CuO, (**d**) Cu2p of PBA-GO-CuO.

We performed XPS tests to understand the diverse valence states of boron doping and the variation in the element composition of PBA-GO-CuO nanomaterials, as shown in Figure 3. The curve fitting and analysis of C_1s_ and B_1s_ signals are presented in Table 1. The C_1s_ peak at 290.2 eV, O_1s_ peak at 530.9 eV, and the peak at 195.2 eV is related to the B_1s_ peak in Figure 3a. The high-resolution C_1s_ spectrum of PBA-GO-CuO consists of five characteristic peaks in Figure 3b, corresponding to C–B (288.3 eV), C–C (289 eV), C–O (291.2 eV), C=O (292.5 eV), and O–C=O (287.5 eV) structures. The high-resolution B_1s_ peak was synthesized into four peaks (Figure 3c), representing the structures of B_4_C (187.8 eV, attributed to the graphene lattice defects), BC_3_ (189.9 eV, may indicate that boron atoms replace carbon atoms in the graphene skeleton), BC_2_O (191.2 eV, may suggest that boron atoms replace carbon atoms in the edge or defect position of the graphene skeleton), and BCO_2_ (192.3 eV, same as BC_2_O) [39,40,41,42]. Figure 3d is a high-resolution spectrum of Cu, where the peaks observed at 933.2 and 953.8 eV are due to Cu_2p3/2_ and Cu_2p1/2_, which are attributed to oxidized Cu (II) [41]. 

### 2.2. Electrochemical Characteristics of Modified Electrodes

**Figure 4:**
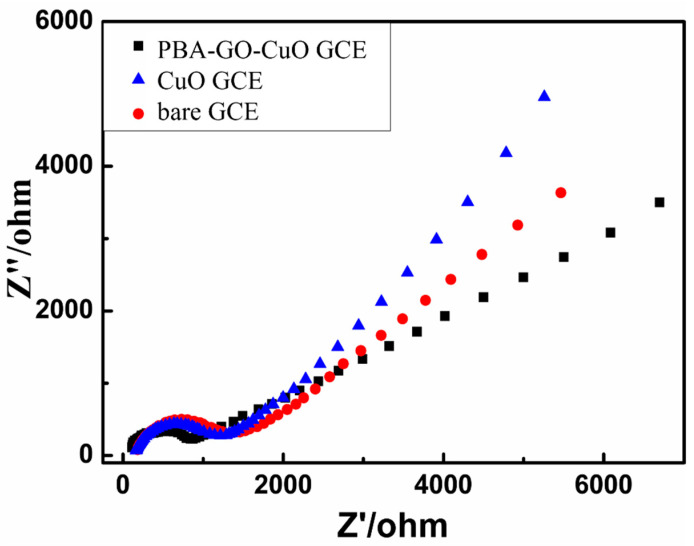
Impedance diagram of bare GCE, CuO-GCE, and PBA-GO-CuO-GCE in 0.1 KCl electrolyte solution containing 5 mM Fe(CN) 6^3−/4−^.

Electrochemical impedance spectroscopy is a valuable method to reflect the interfacial changes in the sensor in which the semicircle portion at a higher frequency expresses the electron-transfer-limited process, and the line at a lower frequency characterizes the diffusion process. The semicircle diameter order in Figure 4 is equal to that of the electron-transfer resistance: bare Glassy Carbon electrode (GCE) > CuO-GCE > PBA-GO-CuO-GCE. PBA-GO-CuO-GCE’s diffusion uniformity (more parallel to the X-axis) is more excellent than other modified materials. In summary, the PBA-GO-CuO-GCE has the best response to glucose.

### 2.3. Electrochemical Response of Modified Electrode to Glucose

**Figure 5:**
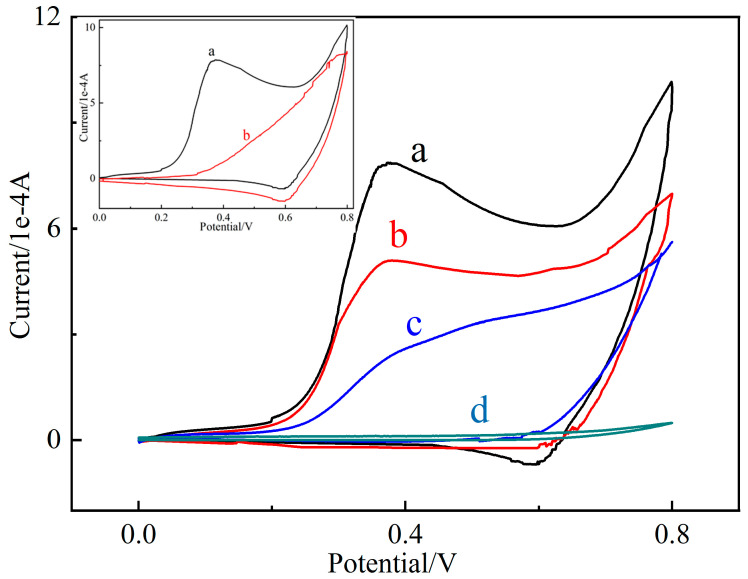
The cyclic voltammograms of bare GCE (**d**), CuO GCE (**c**), GO-CuO-GCE (**b**), and PBA-GO-CuO-GCE (**a**) in 0.1 M NaOH solution with 10 mM glucose (scan rate: 0.4 V/s); inset: PBA-GO-CuO-GCE in 0.2 M NaOH with (**a**) and without (**b**) the injection of 10 mM glucose.

Cu (II)/Cu (III) redox peaks are essential in nonenzymatic electrochemical glucose signal enlargement. As shown in the blue curve in Figure 5, CuO was oxidized to Cu (III) species, including CuO(OH) or another compound at an oxidation peak of about +0.4 V, and the generated Cu (III) species catalyzed the oxidation of glucose to glycoside at a scanning rate of 0.4 V/s. The PBA-GO-CuO-GCE displays poor redox peaks in 0.1 M NaOH without glucose (Figure 5). At the same time, Cu (III) was reduced to Cu (II) at the reduction peak of about +0.6 V. It is evident in Figure 5 that the bare GCE displays an inconspicuous redox peak at 0–0.8 V, which also confirms the signal amplification effect of the copper pair. After added glucose, electrons were quickly transferred from the glucose to the electrode. Cu (III) ions received electrons and functioned as electron-transfer carriers [43]. The black and red curves represent the redox peaks of PBA-GO-CuO-GCE and GO-CuO-GCE, respectively. Additionally, the approximate ratio of the closed-loop area of the four cyclic voltammetry curves in Figure 5 is 2.2: 1.57: 1.04: 0.07. The synergic signal enhancement is due to two reasons. Firstly, the electronic defects of the B element can cause the positively charged PBA-GO to be more likely to function as an electron receiver, absorbing electrons on the electrode; secondly, the CuO deposited on the PBA-GO is uniformly distributed, providing highly catalytically active sites and a high-efficiency glucose oxidation platform.

**Figure 6:**
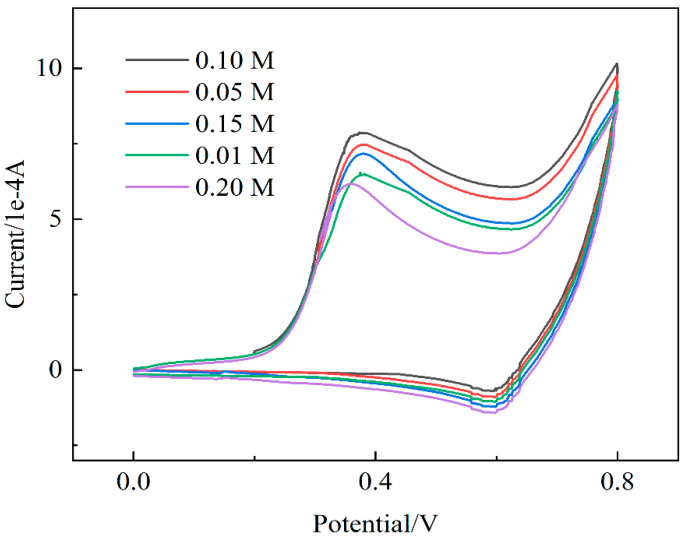
CVs of PBA-GO-CuO-GCE toward 10 mM glucose at 0.4 V under different NaOH concentrations (0.01, 0.05, 0.10, 0.15, 0.20); scan rate: 0.4 mV/s.

Prior to nonenzymatic glucose detection, the alkaline medium may be favorable to improve the electrocatalytic activity of the transition metal-based catalysts. Hence, the impact of NaOH concentrations was investigated in amperometry measurements of 0.1 M glucose. As shown in Figure 6, the amperometry currents increase correspondingly when the electrolyte concentration increases from 0.01 to 0.10 M because glucose is more easily oxidized, and the electrocatalytic activity of NN-CuO is greatly enhanced at high OH-. However, the peak current is decreased by further increasing the electrolyte concentration from 0.10 to 0.20 M. A possible reason may be that too much OH- can block the further electro-adsorption of glucose anion and result in a decrease in the current signal.

### 2.4. Chronoamperometry Studies

**Figure 7:**
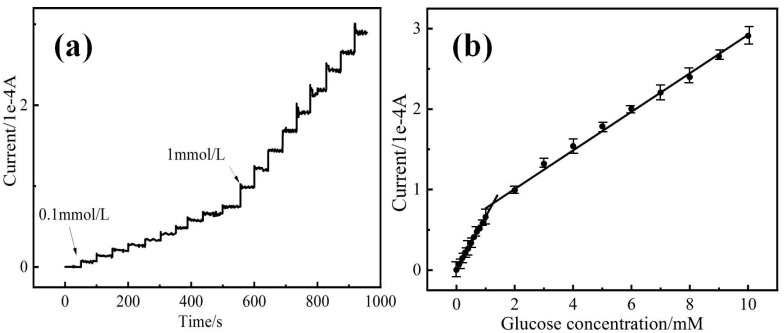
(**a**) I-t response of the PBA-GO-CuO electrode recorded at 0.4 V with the successive addition of an amount of glucose, (**b**) linear relationship between I vs. c of glucose.

The chronoamperometry and a calibration curve of the PBA-GO-CuO-GCE glucose sensor are shown in Figure 7. A stable and fast stair-shape current-time signal responsive diagram can be observed in Figure 7a. In the first portion of the stair diagram, a 0.10 mM glucose solution was repeatedly added into a 0.10 M NaOH electrolyte after every 50 s, resulting in a current increase by 7.1 × 10^−6^ after each operation; in the second part of the stair diagram, a 1 mM glucose solution was repetitively added over 10 times, and the enlarged stair-shape signal occurred. The second part of the current is three times faster than the first. However, the current-time signal noise fluctuates after repeated glucose addition because the intermediate products are overlapped on the electrode due to signal interference [44]. With the continuous increase in glucose concentration of 1.5 mM and 2 mM, the current tends are stable, so the current-time relationship between 0.1 and 10 mM was selected for further study.

As shown in Figure 7b, the calibrated diagram consisted of two linear current–concentration curves as follows: Y_1_ (10^−4^ A) = 0.6432 X_1_ (mM) + 0.0136 (R^2^ = 0.99794) (low concentration range of 0.1–1 mM); Y_2_ (10^−4^ A) = 0.2305 X_2_ (mM) + 0.5951 (R^2^ = 0.99657) (high concentration range of 1–10 mM). The detection limit is 0.7 μΜ (S/N = 3), and the calculated sensitivity is ~906 μA mM^−1^ cm^−2^ and 325 μA mM^−1^ cm^−2^ (the geometrical area and diameter of GCE are 7.068 and 3 mm, respectively). The sensitivity at a high concentration range is less than at a low concentration range, possibly due to the intermediate product generated by the electrocatalytic oxidation of glucose that was absorbed [45]. In contrast, the adsorption kinetics of glucose is slower at high concentrations. The detection performance of the fabricated modified electrode is compared with GO-CuO-GCE, CuO-GCE, and some other GCE-based nonenzymatic sensors. As can be seen in Table 2, the PBA-GO-CuO-GCE-modified electrode has a lower detection limit and a wider linear range [45,46,47,48,49,50,51,52,53,54,55,56].

### 2.5. Ultrasensitive Screening of Inhibitors from Natural Products

**Figure 8:**
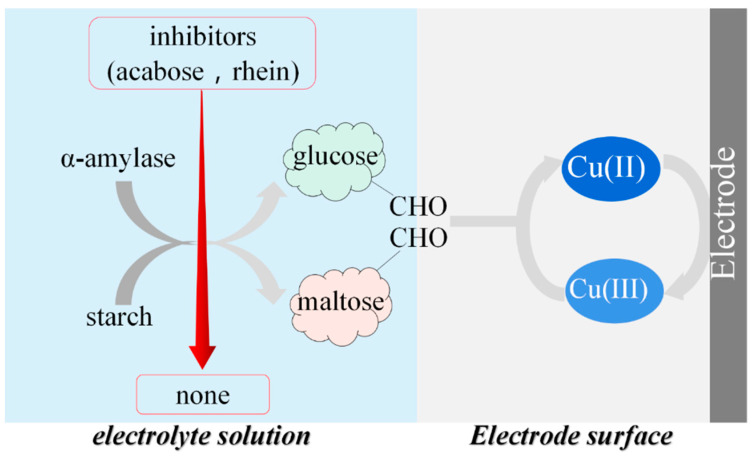
Schematic diagram of the mechanism of the method.

**Figure 9:**
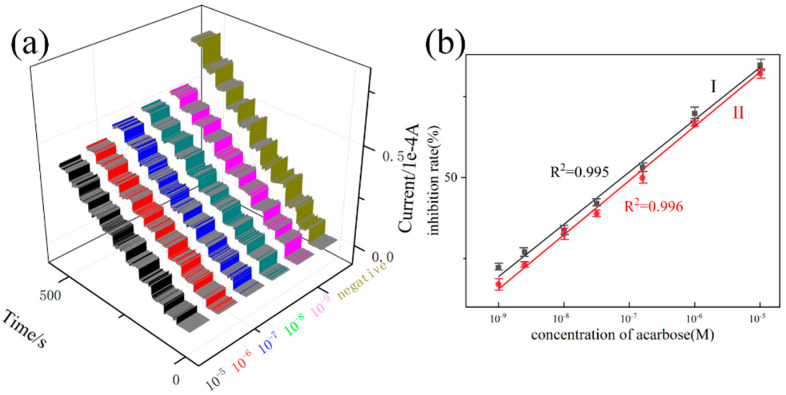
(**a**) The current-time curve with acarbose concentration of 1.0 × 10^−9^ M, 5.0 × 10^−8^ M, 1.0 × 10^−8^ M, 5.0 × 10^−7^ M, 1.0 × 10^−7^ M, 1.0 × 10^−6^ M, 1.0 × 10^−5^ M, (**b**) I: electrochemical standard curve; II: iodine assay colorimetric method standard curve.

**Figure 10:**
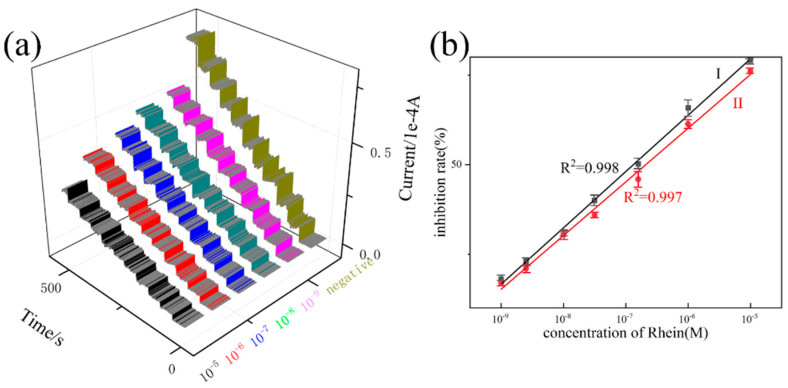
(**a**) The current-time curves with Rhein concentrations of 1.0 × 10^−9^ M, 5.0 × 10^−8^ M, 1.0 × 10^−8^ M, 5.0 × 10^−7^ M, 1.0 × 10^−7^ M, 1.0 × 10^−6^ M, 1.0 × 10^−5^ M, (**b**) I: electrochemical standard curve; II: iodine assay colorimetric standard curve.

The established glucose sensor was applied to study the inhibitor of α-amylase, and the inhibiting ability can be sensitivity reported by the current responsive intensity. As described in Figure 8, the process of α-amylase inhibition limits glucose production and ultimately affects the strength of the current response. As one of the commonly used clinical α-amylase inhibitors, acarbose was tested as a positive drug. As shown in Figure 9a, the levels of current responsive intensity relative to the doses of the inhibitors after the inhibitors were added into the α-amylase reaction mixture. Serials of acarbose concentrations (1.0 × 10^−9^ M, 5.0 × 10^−8^ M, 1.0 × 10^−8^ M, 5.0 × 10^−7^ M, 1.0 × 10^−7^ M, 1.0 × 10^−6^ M, 1.0 × 10^−5^ M) were tested with a linear equation of I (%) = 919.426 C_glu_ + 11.602 and a good correlation coefficient of 0.997. The developed platform provided a detection of 0.95 nM at a signal-to-noise ratio of 3, indicating ultra-sensitivity. By calculating the regression equation, the IC_50_ values are 48.6 μM. In order to further prove the applicability of the developed method, five compounds belonging to flavonoids (which was accomplished through our protein hybrid nanoflower technology) were screened [36]. The results of the Rhein-8-b-D-glucopyranoside are shown in Figure 10a. It can be seen that our method can efficiently detect the inhibition ability driven by Rhein-8-b-D-glucopyranoside. The good linear correlations of I (%) = 1014.056 C_Rhe_ + 216.239(R^2^ = 0.997) were obtained for Rhein-8-b-D-glucopyranoside, and its limit of detection (LOD) was 1.39 nM. By calculating the regression equation, the IC_50_ value of Rhein-8-b-D-glucopyranoside is 39.1 μM. The IC_50_ values of acarbose (48.6 μM), Rhein-8-b-D-glucopyranoside (39.1 μM), indicated that Rhein-8-b-D-glucopyranoside possessed the most powerful inhibiting activity followed by acarbose. Moreover, the inhibiting ability of acarbose and Rhein-8-b-D-glucopyranoside were also investigated by the Iodine assay colorimetric method, and results are shown in Figure 9b and Figure 10b [37]. The above results of the iodine assay colorimetric method powerfully demonstrate that the new sensing platform is capable of screening α-amylase. Because α-amylase is an important target in diabetes, these results further verified that the natural medicines containing Rhein-8-b-D-glucopyranoside often possess anti-diabetes activity.

### 2.6. Sensor Repeatability, Selectivity, and Stability

Superior repeatability and stability are critical factors in measuring electrode preparation success. We examine the stability of the electrode from the following two aspects. First, five freshly prepared PBA-GO-CuO electrodes were used to measure a 0.2 M NaOH solution (adding 10.0 mM glucose). The relative standard deviation of the electrode is 2.8% (*n* = 5), indicating that the designed and prepared PBA-GO-CuO electrode has excellent repeatability. Then, one of the electrodes was tested five consecutive times and washed with distilled water after each test. The relative standard deviation of the measured oxidation peak was 2.6%. In conclusion, the PBA-GO-CuO electrode prepared using this design method has good repeatability.

Moreover, the prepared PBA-GO-CuO electrode was stored in the dark for 2 months. After 2 months, it was used to measure a 0.2 M NaOH solution (10.0 mM glucose added). The oxidation current peak remained at 96%, signifying that the PBA-GO-CuO electrode prepared using this design method has excellent stability.

Additionally, some interfering ions, starch, inhibitor, and components in herbal medicine which may be present in the electrolyte solution were used to influence the determination results. It was observed that the tested substances had no practical influence on our detection platform. The high selectivity of the developed method is due to the specific response of the detection platform.

## 3. Materials and Methods

### 3.1. Materials and Apparatus

Graphene oxide aqueous solution (GOs-325, 2 mg/mL, ≥99.9%), 1-pyreneboronic acid (PBA), copper (II) acetate monohydrate, acarbose (98%), α-amylase (from porcine pancreas, type VI-B, ≥10 units/mg), soluble starch, potassium hexacyanoferrate (II) trihydrate, potassium hexacyanoferrate (III), D-(+)-glucose, and Nafion dispersion solution were purchased from Sigma-Aldrich (Sigma-Aldrich, Shanghai Titan Technology Co., Ltd., Shanghai, China). Rhubarb was purchased from Jiangxi Zhihetang Chinese Medicine Decoction Pieces Co., Ltd. (Jiangxi, China, batch numbers 160501 and 160801).

Electrochemical measurements were tested by a CHI800D electrochemical workstation (Shanghai Chenhua Instrument Co., Ltd., Shanghai, China). All electrochemical experiments were carried out on a three-electrode system, including a bare or modified GCE as the working electrode (WE)) (prior to surface coating, the GCE was polished carefully with 1.0, 0.3, and 0.05 μm alumina powder, respectively. Then, the polished GCE was cleaned sequentially with 1:1 HNO_3_, ethanol, and water by continuous sonication, respectively. The electrode was allowed to dry at ambient temperature for further use). A Pt piece electrode was used as the counter electrode (CE), and an Ag/AgCl (3 M KCl) as the reference electrode (RE). All electrodes were purchased from Sigma-Aldrich (Sigma-Aldrich Company, Shanghai, China). Transmission electron microscopy (TEM) images were obtained by a Hitachi HT7700 (Shanghai, China), scanning electron microscopy (TEM) images were recorded by a JEOL JSM-6700 (Shanghai, China), X-ray photoelectron spectroscopy (XPS) images were recorded by a PHI5000V VersaProbe (Shanghai, China), and Fourier transform infrared (FTIR) images were recorded by a Nicolet Avatar 370 spectrometer (Jiangsu Skyray instruments Co., Ltd., Shanghai, China).

### 3.2. Preparation of PBA-GO-CuO Nanocomposite

CuO, GO-CuO, and PBA-GO-CuO were synthesized according to the reported studies with minor revisions to boron doping [40]. Then, 175 mg of a 5 mg/mL PBA was added into 35 mL of a 1.5 mg/mL GO suspension by intense agitation for 60 min. Then, the amount of Cu (CH_3_COO)_2_ H_2_O was added dropwise to allow the copper source to adsorb on the graphene oxide; subsequently, the as-obtained turbid suspension was transferred into a high-temperature high-pressure autoclave and subjected to the hydrothermal reduction at 180 °C for 12 h. For comparison experiments, both CuO (without adding PBA and GO, the other steps remained the same) and GO-CuO (replaced PBA and GO with GO, the other steps remained the same) were synthesized according to a similar procedure. Afterward, the resulting dark precipitates were collected by centrifugation and washed with deionized water. Finally, the purified precipitate was freeze-dried overnight, and the CuO, GO-CuO, and PBA-GO-CuO were obtained for further characterization and preparation of the sensor.

### 3.3. Preparation and Measurement of the Glucose Sensor

The modified glassy carbon electrode (GCE) was formed through a conventional technique. In simple terms, the above-collected PBA-GO-CuO was hand-ground and dispersed with alcohol to obtain a uniform 5 mg/mL solution. A total of 10 μL of dispersion was dropped on the surface of a clean GCE and dried at room temperature. Subsequently, 5 μL of 0.1% Nafion solution was dropped on the surface of a GCE and dried at room temperature to obtain a PBA-GO-CuO-modified electrode, which we labeled PBA-GO-CuO-GCE. CuO, GO, and GO-CuO were prepared using a similar procedure for comparison.

### 3.4. Trace α-Amylase Inhibitor Screening from Natural Product

As a typical α-amylase inhibitor, acarbose was employed to verify the feasibility of the α-amylase inhibitor screening platform. Five natural compounds (i.e., aloe-emodin-8-O-b-D-glucopyranoside, 6-O-cinnamamoyglucose, L-epicatechin, 2-O-cinnamoyl-1-O-galloy-b-D-glucose, and Rhein-8-b-D-glucopyranoside) were also screened. Different concentrations of the tested compounds were prepared by ethanol-water solution (70:30, *v/v*). The assay for α-amylase inhibitors was as follows: (1) 0.5 mL of α-amylase (0.1 U/mL) was incubated in Phosphate Buffer (PBS, pH = 6.8) with different doses of inhibitors, then, 5 mL 30 g/L soluble starch was added dropwise at 37 °C for 20 min, and dried with a flow of N_2_. The blank group, negative control group, and positive control group were prepared using a similar procedure; the glucose content of each reaction system was detected by a glucose sensor. (2) According to the following calculation formula (Equation (1)) for inhibition rate, the inhibition rate of each inhibitor and the positive drug acarbose on α-amylase can be calculated.

(1) $$I/\%=\frac{C_{B}-C_{D}}{C_{B}-C_{A}}$$

where *I* is the enzyme inhibition rate (%), *C_A_* is the glucose concentration (M) measured in the blank group, *C_B_* is the glucose concentration (M) measured in the negative control group, and *C_D_* is the glucose concentration (M) measured in the sample group. The IC_50_ was calculated from the inhibition rate-concentration curve.

## 4. Conclusions

In summary, we successfully synthesized a glucose sensor consisting of a PBA-GO-CuO nanocomposite where the boron doping rate remarkably reaches up to 9.6%, and the CuO load is ~12.5 wt.%, leading to rich pore structure and high specific surface area. This sensor acquires an enhanced signal amplification effect, a more comprehensive linear range (0.1–10 mM), lower detection limit (0.7 μΜ), higher sensitivity (906 μA mM^−1^ cm^−2^), and lower detection potential (+0.4 V). The prepared sensor application in acarbose and Rhein’s inhibitory activity measurer has the advantages of easy preparation, charting, and convenience, providing a reference value and feasible basis for electrochemistry in the additional calculation and activity verification of traditional Chinese medicine.

## Figures and Tables

**Table 1:** XPS elemental analysis of PBA-GO-CuO.

Element	Species	Binding Energy(eV)	Relative Intensity (%)
C 1s	C-C/C=C	284.01	71.26
	C-N	284.62	18.30
	C-C=O	288.50	5.29
	C=O	287.07	3.08
	C-B	284.81	2.07
B 1s	BC3	189.92	67.21
	BC2O	191.04	18.26
	BCO2	192.13	8.25
	B4C	187.81	6.34

**Table 2:** Comparison of detecting performance of the B-GO-CuO with other nonenzymatic glucose sensors.

Electrode Material	Electrode	Doping Element (and Its Source)	Sensitivity (μA mM^−1^ cm^−2^)	Linear Range	Detection Limit (μM)	Reference
PBA-GO-CuO	GCE	B (1-Pyrene boric acid)	906	0.1 mM–2.0 mM	0.7 μM	This work
GO-CuO	GCE	-	723	0.1 mM–2.0 mM	1.5 μM	This work
CuO	GCE	-	206	0.1 mM–2.0 mM	9.5 μM	This work
Faceted CuO nanoribbons	GCE	-	412	0.05 mM–3.5 mM	58 μM	Sahoo et al. [46]
LSC/rGO	GCE	-	330	2 μM–3.35 mM	63 μM	He et al. [47]
GO/CuO	GCE	-	37.63	0.005 mM–14 mM	5.04 μM	Foroughi et al. [48]
MWCNT/Au	GCE/CSPE	-	2.77 ± 0.14	0.1 mM–20 mM	4.1 μM	Branagan et al. [49]
Au/Cu_2_O/GCE	GCE	-	715	0.05 mM–2.0 mM	18 μM	Su et al. [50]
Co-MOF Nanosheets	GCE	-	219.67	0.5 μM–8.065 mM	0.25 μM	Li et al. [51]
Co/MoS_2_/CNTs	GCE	-	131.69	0–5.2 mM	80 nM	Branagan et al. [49]
Ni(II)-CP/C_60_	GCE	-	614.29	0.01 mM–3.00 mM	4.3 μM	Shahhoseini et al. [52]
3D flower-like Ni_7_S_6_	GCE	-	271.8	5 μM–3.7 mM	0.15 μM	Wu et al. [53]
N-GR- CNTs/AuNPs	GCE	N(HNO_3_)	0.9824	2 μM–19.6 mM	0.5 μM	Jeong et al. [55]
Microwave N-GO/CuO	GCE	N(urea)	122.336	0.01 mM–10 mM	14.52 μM	Rahsepar et al. [31]
S-rGO/CuS	GCE/RDE	S(Na_2_S)	429.4	3.88 mM–20.17 mM	0.032 μM	Karikalan et al. [56]

## Data Availability

Not applicable.

## Sample Availability

Samples of the compounds are not available from the authors.

## References

[1] Liu L.L., Cen Y., Liu F., Yu J.G., Jiang X.Y., Chen X.Q. (2015). Analysis of α-amylase inhibitor from corni fructus by coupling magnetic cross-linked enzyme aggregates of α-amylase with HPLC-MS. J. Chromatogr. B.

[2] Malene J.P., Rita D.C., Louise K., Dan S. (2019). Immobilized α-amylase magnetic beads for ligand fishing: Proof of concept and identification of α-amylase inhibitors in Ginkgo biloba. Phytochemistry.

[3] Lima H.R., Silva J., Farias E. (2018). Electrochemical sensors and biosensors for the analysis of antineoplastic drugs. Biosens. Bioelectron..

[4] Mourzina Y.G., Ermolenko Y.E., Offenhäusser A. (2021). Synthesizing Electrodes into Electrochemical Sensor Systems. Front. Chem..

[5] Ye Y.L., Ji J., Sun Z.Y. (2019). Recent advances in electrochemical biosensors for antioxidant analysis in foodstuff. TrAC Trends Anal. Chem..

[6] Cortina M.E., Melli L.J., Roberti M. (2016). Electrochemical magnetic microbeads-based biosensor for point-of-care serodiagnosis of infectious diseases. Biosens. Bioelectron..

[7] Lv M., Liu Y., Geng J.H. (2018). Engineering nanomaterials-based biosensors for food safety detection. Biosens. Bioelectron..

[8] Zhang X.L., Wu D., Zhou X.X. (2019). Recent progress on the construction of nanozymes-based biosensors and their applications to food safety assay. TrAC Trends Anal. Chem..

[9] Silva N.F., Magalhães J.M., Freire C. (2018). Electrochemical biosensors for Salmonella: State of the art and challenges in food safety assessment. Biosens. Bioelectron..

[10] Hovancová J., Šišoláková I., Oriňaková R. (2017). Nanomaterial-based electrochemical sensors for detection of glucose and insulin. J. Solid-State Electrochem..

[11] Tajik S., Beitollahi H., Ahmadi S.A. (2021). Screen-printed electrode surface modification with NiCo2O4/RGO nanocomposite for hydroxylamine detection. Nanomaterials.

[12] Beitollahi H., Shahsavari M., Sheikhshoaie I. (2022). Amplified electrochemical sensor employing screen-printed electrode modified with Ni-ZIF-67 nanocomposite for high sensitive analysis of Sudan I in present bisphenol A. Food Chem. Toxicol..

[13] Adeel M., Rahman M.M., Caligiuri I. (2020). Recent advances of electrochemical and optical enzyme-free glucose sensors operating at physiological conditions. Biosens. Bioelectron..

[14] Han T., Noda S., Haneda K. (2018). Development of a glucose sensor using palladium electrode. Diabetes.

[15] Zhu H., Li L., Zhou W. (2016). Advances in non-enzymatic glucose sensors based on metal oxides. J. Mater. Chem. B.

[16] Qian J.C., Wang Y.P., Pan J. (2020). Non-enzymatic glucose sensor based on ZnO–CeO_2_ whiskers. MCP.

[17] Sode K.J., Loew N.Y., Ohnishi Y.S. (2017). Novel fungal FAD glucose dehydrogenase derived from Aspergillus niger for glucose enzyme sensor strips. Biosens. Bioelectron..

[18] Lee H., Hong Y.J., Baik S. (2018). Enzyme-based glucose sensor: From invasive to wearable device. Adv. Healthc. Mater..

[19] Chen D., Wang X.H., Zhang K.X. (2020). Glucose photoelectrochemical enzyme sensor based on competitive reaction of ascorbic acid. Biosens. Bioelectron..

[20] Salazar P., Rico V., Rodríguez-Amaro R. (2015). New Copper wide range nanosensor electrode prepared by physical vapor deposition at oblique angles for the non-enzimatic determination of glucose. Electrochim. Acta.

[21] Liu L., Wang M., Wang C.Y. (2018). In-situ synthesis of graphitic carbon nitride/iron oxide−copper composites and their application in the electrochemical detection of glucose. Electrochim. Acta.

[22] Duan X.X., Liu K.L., Xu Y. (2019). Nonenzymatic electrochemical glucose biosensor constructed by NiCo_2_O_4_@Ppy nanowires on nickel foam substrate. Sens. Actuators B Chem..

[23] Wei M., Qiao Y.X., Zhao H.T. (2020). Electrochemical non-enzymatic glucose sensors: Recent progress and perspectives. ChemComm.

[24] Sun C., Miao J.J., Yan J. (2013). Applications of antibiofouling PEG-coating in electrochemical biosensors for determination of glucose in whole blood. Electrochim. Acta.

[25] Wang J., Zhang W.D. (2011). Fabrication of CuO nanoplatelets for highly sensitive enzyme-free determination of glucose. Electrochim. Acta.

[26] Fleischmann M., Korinek K., Pletcher D. (1972). The kinetics and mechanism of the oxidation of amines and alcohols at oxide-covered nickel, silver, copper, and cobalt electrodes. J. Chem. Soc. Perkin Trans..

[27] Seunghee H.C., Sun S.K., Jaeseok Y. (2016). Chemical and biological sensors based on defect-engineered graphene mesh field-effect transistors. Nano Converg..

[28] Zhang Y.P., Liu X.T., Qiu S. (2019). A Flexible Acetylcholinesterase-Modified Graphene for Chiral Pesticide Sensor. J. Am. Chem. Soc..

[29] Sehit E., Altintas Z. (2020). Significance of nanomaterials in electrochemical glucose sensors: An updated review (2016–2020). Biosens. Bioelectron..

[30] Wei S.J., Hao Y.B., Ying Z. (2020). Transfer-free CVD graphene for highly sensitive glucose sensors. J. Mater. Sci. Technol..

[31] Rahsepar M., Foroughi F., Kim H. (2019). A new enzyme-free biosensor based on nitrogen-doped graphene with high sensing performance for electrochemical detection of glucose at biological pH value. Sens. Actuators B Chem..

[32] Paraknowitsch J.P., Thomas A. (2013). Doping carbons beyond nitrogen: An overview of advanced heteroatom doped carbons with boron, sulphur and phosphorus for energy applications. Energy Environ. Sci..

[33] Li Y.F., Zhang W.W., Guo B. (2017). Interlayer shear of nanomaterials: Graphene-graphene, boron nitride-boron nitride and graphene-boron nitride. Acta Mech. Solida Sin..

[34] Yu X.M., Han P., Wei Z.X. (2018). Boron-Doped Graphene for Electrocatalytic N2 Reduction. Joule.

[35] Justino C., Gomes A.R., Freitas A.C. (2017). Graphene based sensors and biosensors. Trac-Trend Anal. Chem..

[36] Qian K., Wang H., Liu J.M. (2017). Synthesis of α-glycosidase hybrid nano-flowers and their application for enriching and screening α-glycosidase inhibitors. New J. Chem..

[37] Xie Y.S., Li D.W., Li Y.F. (2015). The activity of α-amylase inhibitor was determined by colorimetric method of iodine test solution. Jiangsu Agric. Sci..

[38] Rotte N.K., Naresh V., Muduli S. (2020). Microwave aided scalable synthesis of sulfur, nitrogen co-doped few-layered graphene material for high-performance supercapacitors. Electrochim. Acta.

[39] Jiang B., Liang K.M., Yang Z.J. (2021). FeCoNiB@Boron-doped vertically aligned graphene arrays: A self-supported electrocatalyst for overall water splitting in a wide pH range. Electrochim. Acta.

[40] Zhou X.Y., Zhang J., Su Q.M. (2014). Nanoleaf-on-sheet CuO/graphene composites: Microwave-assisted assemble and excellent electrochemical performances for lithium-ion batteries. Electrochim. Acta.

[41] Gao J.P., Qiu G.J., Li H.J. (2020). Boron-doped graphene/TiO2 nanotube-based aqueous lithium-ion capacitors with high energy density. Electrochim. Acta.

[42] Shumba M., Nyokong T. (2016). Electrode modification using nanocomposites of boron or nitrogen doped graphene oxide and cobalt (II) tetra aminophenoxy phthalocyanine nanoparticles. Electrochim. Acta.

[43] Yang S.L., Li G., Wang D. (2017). Synthesis of nanoneedle-like copper oxide on N-doped reduced graphene oxide: A three-dimensional hybrid for nonenzymatic glucose sensor. Sens. Actuators B Chem..

[44] Li Y.R., Wang X., Yang Q. (2017). Ultra-fine CuO Nanoparticles Embedded in Three-dimensional Graphene Network Nanostructure for High-performance Flexible Supercapacitors. Electrochim. Acta.

[45] Yadav H.M., Lee J.J. (2019). One-pot synthesis of copper nanoparticles on glass: Applications for non-enzymatic glucose detection and catalytic reduction of 4-nitrophenol. J. Solid State Electrochem..

[46] Sahoo R.K., Das A., Samantaray K. (2019). Electrochemical glucose Sensing characteristics of two-dimensional faceted and non-faceted CuO nanoribbons. Crystengcomm.

[47] He J., Sunarso J., Zhu Y.L. (2017). High-performance non-enzymatic perovskite sensor for hydrogen peroxide and glucose electrochemical detection. Sens. Actuators B Chem..

[48] Foroughi F., Rahsepar M., Hadianfard M.J., Kim H. (2017). Microwave-assisted synthesis of graphene modified CuO nanoparticles for voltammetric enzyme-free sensing of glucose at biological pH values. Microchim. Acta.

[49] Branagan D., Breslin C.B. (2019). Electrochemical detection of glucose at physiological pH using gold nanoparticles deposited on carbon nanotubes. Sens. Actuators B Chem..

[50] Su Y., Guo H., Wang Z.S. (2018). Au@Cu_2_O core-shell structure for high sensitive non-enzymatic glucose sensor. Sens. Actuators B Chem..

[51] Li Q., Shao Z.F., Han T. (2019). A High-Efficiency Electrocatalyst for Oxidizing Glucose: Ultrathin Nanosheet Co-Based Organic Framework Assemblies. ACS Sustain. Chem. Eng..

[52] Shahhoseini L., Mohammadi R., Ghanbari B. (2019). Ni (II) 1D-coordination polymer/C60-modified glassy carbon electrode as a highly sensitive non-enzymatic glucose electrochemical sensor. Appl. Surf. Sci..

[53] Wu W.Q., Li Y.B., Jin J.Y. (2016). A novel nonenzymatic electrochemical sensor based on 3D flower-like Ni7S6 for hydrogen peroxide and glucose. Sens. Actuators B Chem..

[54] Zhang Y., Wang L., Yu J. (2016). Three-dimensional macroporous carbon supported hierarchical ZnO-NiO nanosheets for electrochemical glucose sensing. J. Alloys Compd..

[55] Jeong H., Nguyen D.M., Lee M.S. (2018). N-doped graphene-carbon nanotube hybrid networks attaching with gold nanoparticles for glucose non-enzymatic sensor. Sci. Eng. Compos. Mater..

[56] Karikalan N., Karthik R., Chen S.M. (2017). Sonochemical Synthesis of Sulfur Doped Reduced Graphene Oxide Supported CuS Nanoparticles for the Non-Enzymatic Glucose Sensor Applications. Sci. Rep..

